# Diagnostics for Statistical Variable Selection Methods for Prediction of Peptic Ulcer Disease in *Helicobacter pylori* Infection

**DOI:** 10.4172/jpb.1000308

**Published:** 2014-03-28

**Authors:** Hyunsu Ju, Allan R Brasier, Alexander Kurosky, Bo Xu, Victor E Reyes, David Y Graham

**Affiliations:** 1 Departments of Preventive Medicine and Community Health, University of Texas Medical Branch (UTMB), Galveston, TX, USA; 2 Sealy Center for Molecular Medicine, University of Texas Medical Branch, Galveston, TX, USA; 3 Institute for Translational Sciences, University of Texas Medical Branch, Galveston, TX, USA; 4 Departments of Medicine, Michael E. DeBakey Veterans Affairs Medical Center, Houston, TX 77030, USA; 5 Departments of Biochemistry and Molecular Biology, University of Texas Medical Branch, Galveston, TX, USA; 6 Department of Pediatrics, University of Texas Medical Branch, Galveston, TX, USA; 7 Department of Microbiology & Immunology, University of Texas Medical Branch, Galveston, TX, USA; 8 Baylor College of Medicine, Houston TX, USA

**Keywords:** Variable selection, Classification, Amino acid analysis, Peptic ulcer disease, *Helicobacter pylori*

## Abstract

**Background:**

The development of accurate classification models depends upon the methods used to identify the most relevant variables. The aim of this article is to evaluate variable selection methods to identify important variables in predicting a binary response using nonlinear statistical models. Our goals in model selection include producing non-overfitting stable models that are interpretable, that generate accurate predictions and have minimum bias. This work was motivated by data on clinical and laboratory features of *Helicobacter pylori* infections obtained from 60 individuals enrolled in a prospective observational study.

**Results:**

We carried out a comprehensive performance comparison of several nonlinear classification models over the *H. pylori* data set. We compared variable selection results by Multivariate Adaptive Regression Splines (MARS), Logistic Regression with regularization, Generalized Additive Models (GAMs) and Bayesian Variable Selection in GAMs. We found that the MARS model approach has the highest predictive power because the nonlinearity assumptions of candidate predictors are strongly satisfied, a finding demonstrated via deviance chi-square testing procedures in GAMs.

**Conclusions:**

Our results suggest that the physiological free amino acids citrulline, histidine, lysine and arginine are the major features for predicting *H. pylori* peptic ulcer disease on the basis of amino acid profiling.

## Introduction

The analysis of high-dimensional data, where the number of predictors exceeds the sample size, poses many challenges for statisticians and calls for new statistical methodologies in order to select relevant variables in multivariate data, feature selection is used to overcome the curse of dimensionality by removing non-essential variables to achieve a model with predictive accuracy. Consequently, the choice of a variable selection procedure becomes very important for enhancing the ability to generate reproducible findings and generalizable conclusions. In high-dimensional data it is desirable to have parsimonious or sparse representations of prediction models. Since highly complex models are penalized by increased total error, regularization helps reduce complexity in classification by minimizing over-fitting of the training data. We evaluated this by maximizing goodness-of-fit and simultaneously minimizing the number of variables selected.

In this study, we evaluated different models by randomly selecting and withholding the training data to be used later for testing. The area under the receiver characteristic operating curve (ROC) was used as a measure for comparing prediction accuracy based on sensitivity and specificity for both training and test data. In this study discriminative features were identified that associated with *H. pylori* peptic ulcer disease. We found that various free amino acid measurements could be associated with disease outcome. However, many of these variables are highly correlated and which of the factors will result in the most stable classifier is unknown. Here we sought to extend this work by comparing the effects of various feature reduction methods.

## Methods

### Study design

Sera were obtained from patients with documented *H. Pylori* infection undergoing an endoscopic exam for routine medical management. Thirty subjects with proven duodenal ulcer were matched using sera collected during the same time period. Samples were collected after receiving written informed consent as approved by the Institutional Review Board of Baylor College of Medicine, Houston, TX and the study was conducted in accordance with the guidelines of the Helsinki Declaration.

### Amino acid analysis

Serum was precipitated by mixing equal volumes of 7.5% sulfosalicylic acid (SSA) in 0.02N hydrochloric acid (HCl). Aminoelthyl cysteine (AEC) was added as the internal standard in each sample. The precipitate was centrifuged for 15 min at 10,000 × g, and supernatant containing physiological amino acids was saved. The supernatant was quantified for amino acids using a Hitachi L8800 Amino Acid Analyzer. Data was reported as nmol amino acid per 10 μl plasma. Samples were run in duplicate and concentrations varied less than 10% between duplicates. For measurements below the limits of detection, samples were imputed with 1/10 the lower limit of detection for the assay.

### Feature reduction-modeling strategies

In this paper we use the term feature selection methods to refer to identifying the subset of differentially-expressed predictors that are useful and relevant in distinguishing different classes of samples. Similarly, model selection is a process of seeking the model from a set of candidate models that are the best balance between model fit and complexity. Our research goal is to evaluate the various model fittings of increasing data complexity and to find the best models to identify the underlying model by both AIC, BIC, and cross-validation.

### Feature reduction using Significance Analysis of Microarray (SAM)

SAM determines significance by using more robust test statistics and permutations to estimate false discovery rate instead of the conventional “t” distribution level of significance [1]. Efron et al. [2] developed an empirical bayesian approach using non-informative priors and deriving the posterior probability difference for each of the predictors without having to run t-tests or Wilcoxon tests to identify those that were differentially expressed. In some cases, a heuristic approach was investigated for feature selection by integrating correlation, histogram, and other statistical measures. We used the information criterion on the modeling.

Akaike information criterion (AIC) is given by

(1) AIC(M)=-2lnL(M)+2(p(M)+1),

where L(M) is the maximum likelihood function of the parameters in the model M, and p is the number of covariates estimated in the model [3].

Bayesian information criterion (BIC) is given by

(2) BIC(M)=-2lnL(M)+ln(n)×(p(M)+1),

where n is the sample size, M and p are defined as those variables shown in Equation 1 [4,5]. Specifically, Stone [6] showed that the AIC and leave-one out cross validation are asymptotically equivalent.

### Logistic regression with regularization

Logistic modeling has a binary response *y_i_* ∈ {0, 1}, and assumes that

(3) $$Pr(y=1|x)=1/(1+\exp(-x^{T}\;\beta ),$$

Regularized and shrinkage estimation methods such as a LASSO (least absolute shrinkage and selection operator) estimator helps address variable selection and multicollinearity. For a binary response variable and the logistic regression models, the LASSO estimator is estimated by penalizing the negative log-likelihood with the L_1_-norm. The penalty term is chosen by a cross-validation technique to evaluate the out-of-sample negative log-likelihood [7]. The EN (Elastic Net) penalty is designed to simultaneously select strongly correlated variables that combine the L_1_ and L_2_ penalizing terms in the model [8].

The coefficient vector β that minimizes the penalized log-likelihood is

(4) $$\hat{\beta }={\text{argmin}}_{\beta \in Rp}-\sum (y_{i}\log p_{i}+(1-y_{i})\log(1-p_{i}))+\text{Penalty}(\beta ),$$

where *p_i_* = *Pr*(*y* = 1|*x*).

Fan and Li (2001) proposed the Smoothly Clipped Absolute Deviation (SCAD) penalty, which compromises between L_1_ and L_2_, and the L_0_ selection methods. The SCAD penalty deletes small coefficients and keeps large coefficients unshrunken, but sacrifices continuity and stability. The SCAD penalty can provide a smaller bias in coefficient estimation than LASSO because it is bounded as a function of β. The SCAD penalized estimator also has an oracle property [9]. Sparse regression using penalization is one of the most popular tools for analyzing high dimensional data.

### Generalized Additive Models (GAMs) for classification problems

GAMs provide a general frame work moving beyond linearity by allowing nonlinear functions of each of the variables, while maintaining the additive assumption [10]. Logistic regression GAM modeling has a binary response *y_i_* ∈ {0, 1}, and assumes

(5) $$Pr(y=1|x)=1/(1+\exp(-[\beta_{0}+f_{1}(x_{1})+f_{2}(x_{2})+\cdots +f_{p}(x_{p})]),$$

GAM modeling allowed us to fit a nonlinear function to each predictor, so that we could automatically model the nonlinear relationship that standard linear regression will miss. The nonlinear fits can potentially make accurate predictions for the outcome.

### Multivariate Adaptive Regression Splines (MARS)

The MARS method of Friedman [11] is a nonparametric regression method that estimates complex nonlinear relationship by a series of truncated spline functions of the predictors [12]. The basis functions are combined in the MARS model as a weighted sum of

(6) $${\hat{y}}_{i}=a_{0}+\sum_{k=1}^{p}a_{k}B_{k}(x),$$

where ŷ is the response described by the model, a_0_ the coefficient of the constant basis function (intercept), p the total number of basis functions and a_k_, the coefficient of the *k*^th^ basis function *B_k_*(*x*). MARS models use hockey stick basis functions of the form (*x* − *t*)_+_and (*t* − *x*)_+_, with t being the knot. The basis functions in MARS are single truncated spline functions or a product of two or more spline functions for different predictors. The first order MARS model was built without interactions to over-fit the training data. A maximum number of basis functions equal to 30 was used as the stopping criterion. The model was pruned using a ten-fold generalized cross validation. The optimal model was selected based on evaluation of the model complexity and its predictive quantities for the test sets. Software implementation of the MARS model is available in Salford Predictive Modeler version 7.0 from Salford Systems.

### Bayesian variable selection for GAM

Bayesian variable selection is an approach designed to assess the robustness of results, in terms of alternatives, by calculating posterior distribution over coefficients and models. One of the most popular approaches is to assume a spike-and-slab mixture prior for each coefficient, with one component being a narrow spike around the origin that imposes very strong shrinkage on the coefficients and the other component being a wide slab that imposes very little shrinkage on the coefficients [13]. The posterior weights for the spike and the slab can then be interpreted analogously. To select the models of predictors between smoothing nonlinear terms and linear effects, we performed Bayesian variable selection in GAMs implemented in the R package spikeSlabGAM [14]. Bayesian GAMs produce a posterior probability for each possible model in addition to one for each predictor. Using Bayesian GAMs, model uncertainty can be incorporated into conclusions about parameters and predictions. Thus, we have to consider all possible models that fit. Bayesian GAMs can be applied using the R library BMA (http://cran.r-project.org).

### Nonlinear testing procedures

We assessed the linear or non-linear association of binary response variables of selected variables in each model. Investigation of model predictors and their linear association was determined using GAMs. We evaluated the partial residual plot as a diagnostic graphical tool for identifying nonlinear relationship between the response and covariates for generalized additive models [10,12,15]. For each part predictor, we also examined the log-likelihood ratio test p-values, comparing the deviance between the full model and the model without that variable. We calculated the projection (hat) matrix, Cook’s distance, various residuals and the estimated probabilities versus each predictor to evaluate outliers and identify influential points in the models. We used both the change in residual deviance (as in parametric or nonparametric models), and the ROC to compare the performances of the statistical models.

## Results and Discussion

### Descriptive statistics

Sera from 30 subjects with *H pylori* infection, without endoscopy-documented mucosal ulceration, and 30 subjects with *H pylori* infection and peptic ulceration were studied. Concentrations of the free amino acids were measured in each subject. The concentration of each amino acid by peptic ulcer disease (PUD) status is shown in Table 1. The concentrations of taurine (0.15 ± 0.04 no PUD *vs* 0.19 ± 0.08 with PUD, p<0.05), urea (42.29 ± 11.41 no PUD *vs* 54.17 ± 22.42 PUD, p<0.05), glycine (3.97 ± 0.64 no PUD *vs* 4.64 ± 1.09 with PUD, p<0.05), citrulline (0.34 ± 0.1 no PUD vs. 0.54 ± 0.14 with PUD, P<0.001) were significantly different. Of note, all amino acids were elevated in the subjects with PUD, indicating upregulation of the urea cycle.

### Parametric and nonparametric modeling

Our objective was to create a serum biomarker panel of amino acids that predict the occurrence of PUD. Table 2 shows the sparse regression coefficients with the LASSO, EN, and SCAD penalty which contain important variables and the model selected by using a BIC criterion. Because the underlying data structure dictates the selection of an appropriate modeling approach, we analyzed the contributions of parametric (linear) or nonparametric (spline) features using Bayesian variable selection. This method produces a hierarchy of structured model selections for parametric and nonparametric relationships to the PUD outcome for each feature. The posterior probabilities for the linear and spline components are shown in Table 3. From this analysis, the linear and spline component of citrulline and the spline component of histidine were significant (Table 4).

**Figure 1:**
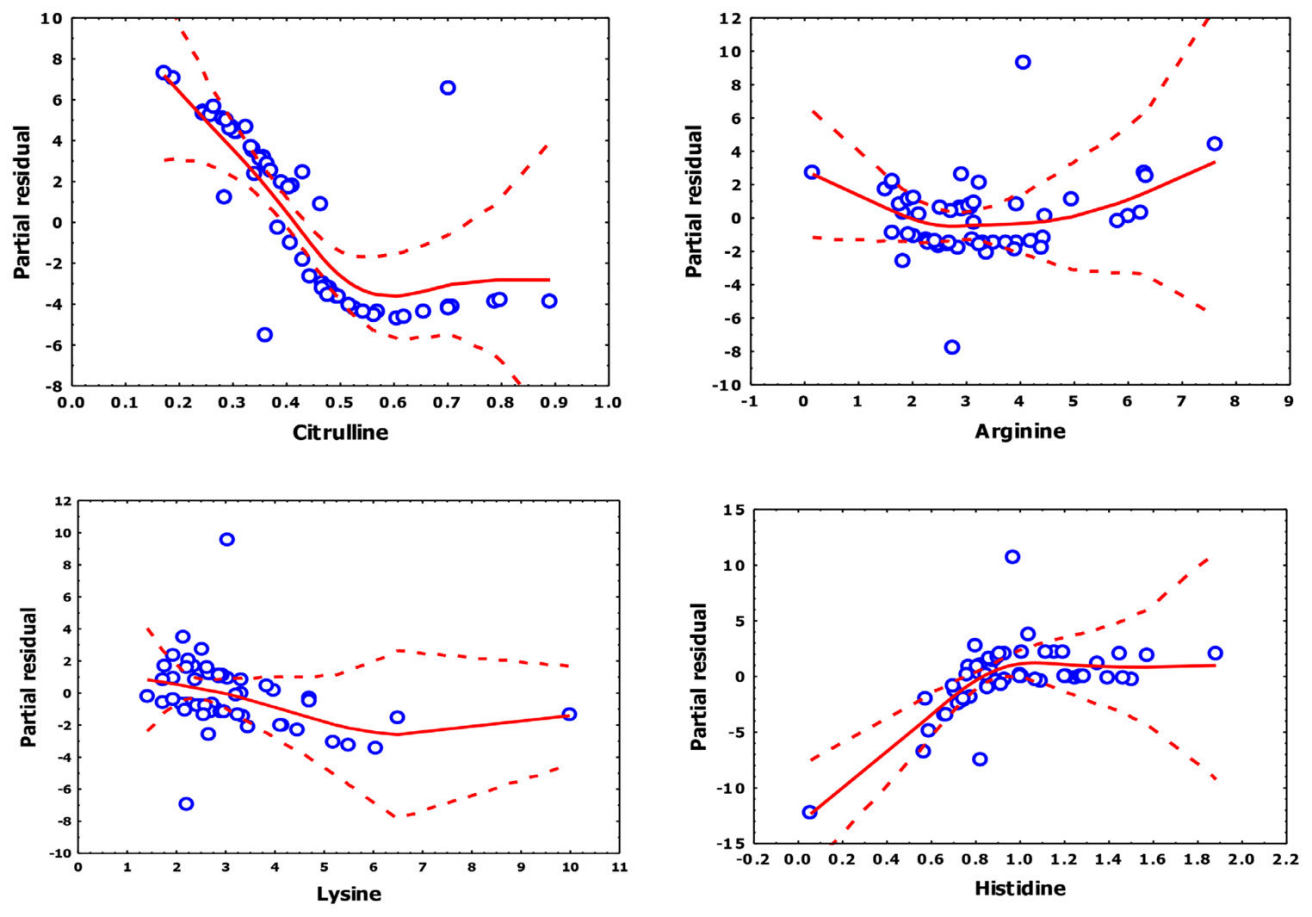
Partial Residual Plots Lines shown are a solid line representing a spline and dotted lines are 95% confidence band for each predictor. For each is shown the relationship between the predictor with residualized (adjusted) dependent variable values.

As an additional analysis, we examined the relationships of amino acids to outcome using GAMs. Inspection of the GAM plots indicates that the partial residuals are nonlinear (Figure 1). For example the partial residual plot of citrulline shows a linear component at low citrulline concentrations until a concentration of 0.5 is reached, at which time the curve sharply inflects to a horizontal line. A similar inflection is seen in other variables. From this analysis, we concluded that the modeling of PUD requires a nonparametric approach.

For the nonparametric modeling we applied MARS, an additive modeling technique that uses piecewise linear spline functions (basis functions) as predictors. MARS uses a two-stage process for constructing the optimal classification model. The first half of the process involves addition of basis functions until a user-specified number of basis functions have been reached. In the second stage, MARS deletes basis functions in order, starting with the basis function that contributes the least to the model until an optimum model is reached.

### Model performance

The optimal MARS identified four informative amino acids (citrulline, histidine, lysine and arginine). Evaluation of the model performance is evaluated in several ways. The accuracy of prediction was evaluated using a confusion matrix. The model produced an overall accuracy of 91.67%, with a 96.67% ability to correctly identify PUD (Table 5).

**Figure 2:**
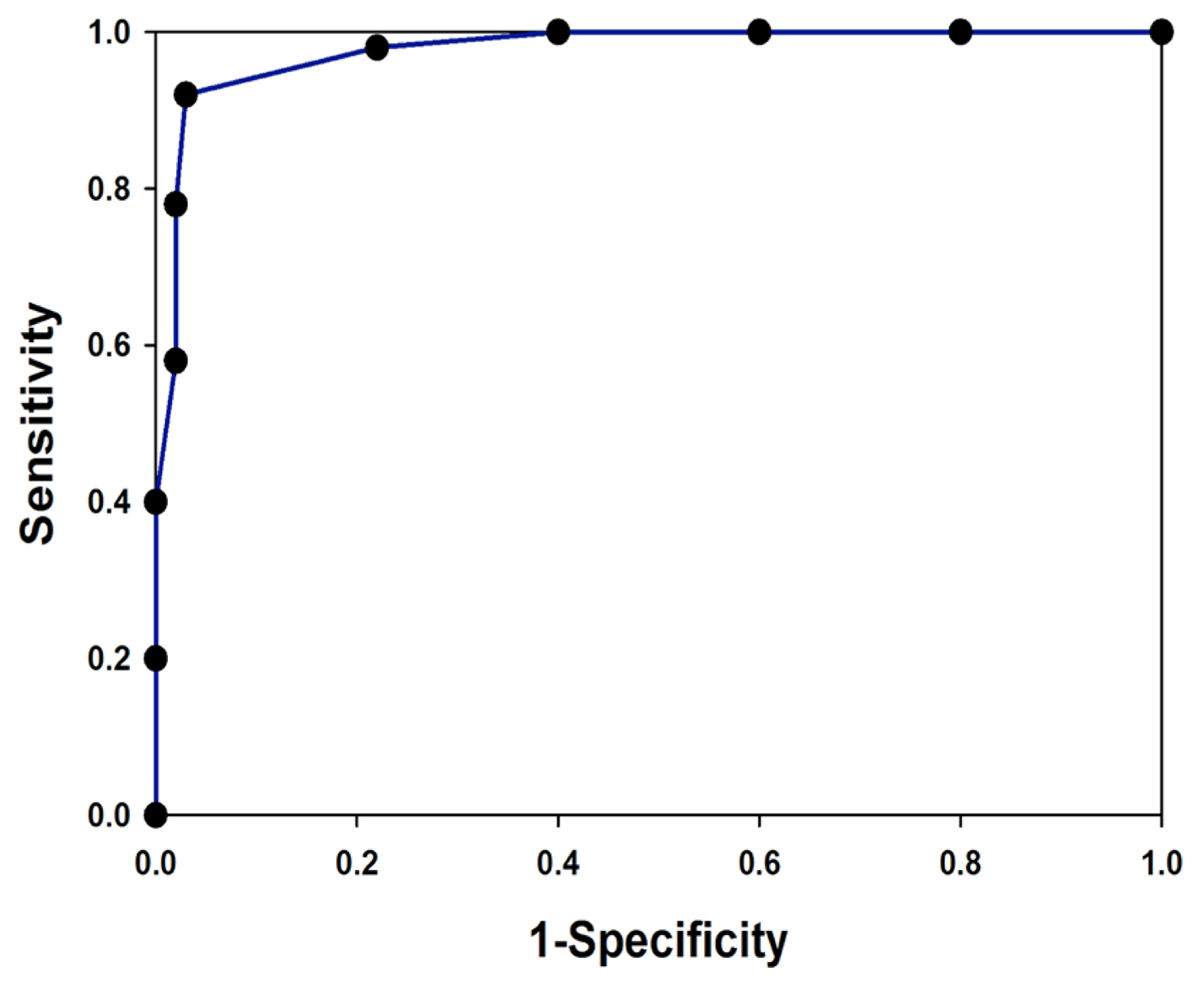
ROC analysis. Shown is a Receiver Operating Characteristic (ROC) curve for the predictive model for peptic ulcer disease. Y axis, Sensitivity; X axis, 1-Specificity.

The second analysis involved evaluation of the ROC, where sensitivity vs. 1-specificity was plotted. In the ROC analysis, a diagonal line (45 degree slope) starting at zero indicates that the output was a random guess, whereas an ideal classifier with a high true positive rate and low false positive rate would curve positively and strongly towards the upper left quadrant of the plot. The AUC is equivalent to the probability that two cases, one chosen at random from each group, are correctly ordered by the classifier. The AUC of the MARS model predictor was 0.9656 (Figure 2), suggesting that the model performed in a highly sensitive and specific manner.

**Figure 3:**
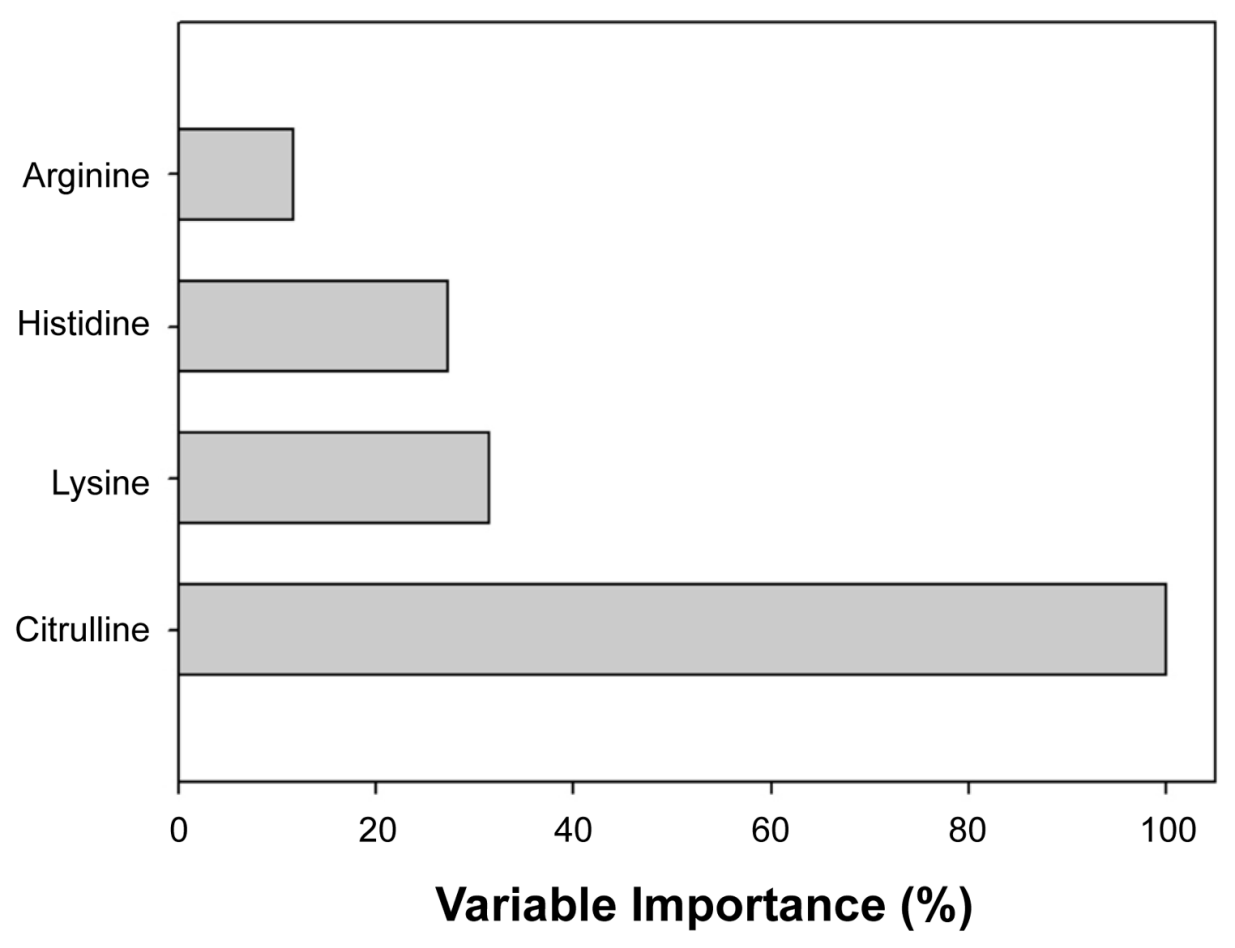
Variable Importance for MARS model of PUD Variable importance was computed for each feature in the MARS model. Y axis, percent contribution for each analyte.

The relative contribution of each amino acid to the overall performance of the classifier was evaluated by the variable importance, a relative measure of the effect of removing a feature on the model accuracy. Here, citrulline was the most important variable (variable importance of 100%), histidine and lysine were less important but similar (31.5% and 27.3%, respectively) and arginine was least important (11.6%, Figure 3).

Finally, the basis functions (BFs), which are combinations of independent variables in the model, are shown in Table 6. Importantly, we note no interaction terms, minimizing the potential for the model to have over-fitted the data. The addition of two BFs for citriulline corresponds well to the inflection of citrulline in the GAM analysis (cf. Figure 1).

**Figure 4:**
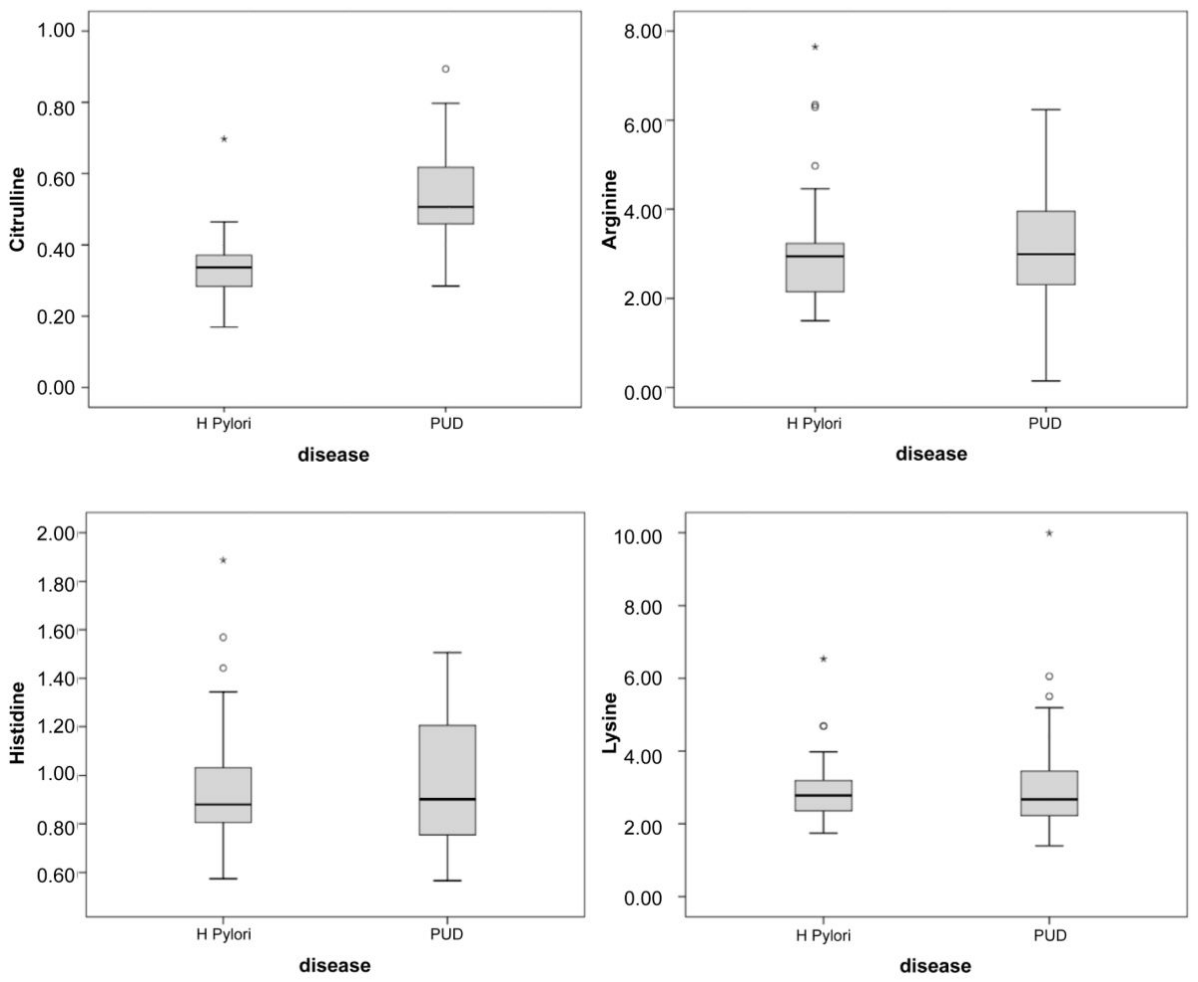
Box Plots For each variable shown, the distribution of each predictor is divided over case (with ulcer) and control (without ulcer). PUD: endoscopy-documented peptic ulcer.

The distributions of concentrations of the amino acids by disease classification are shown in the box plots of Figure 4. A nonparametric relationship is seen for each.

**Figure 5:**
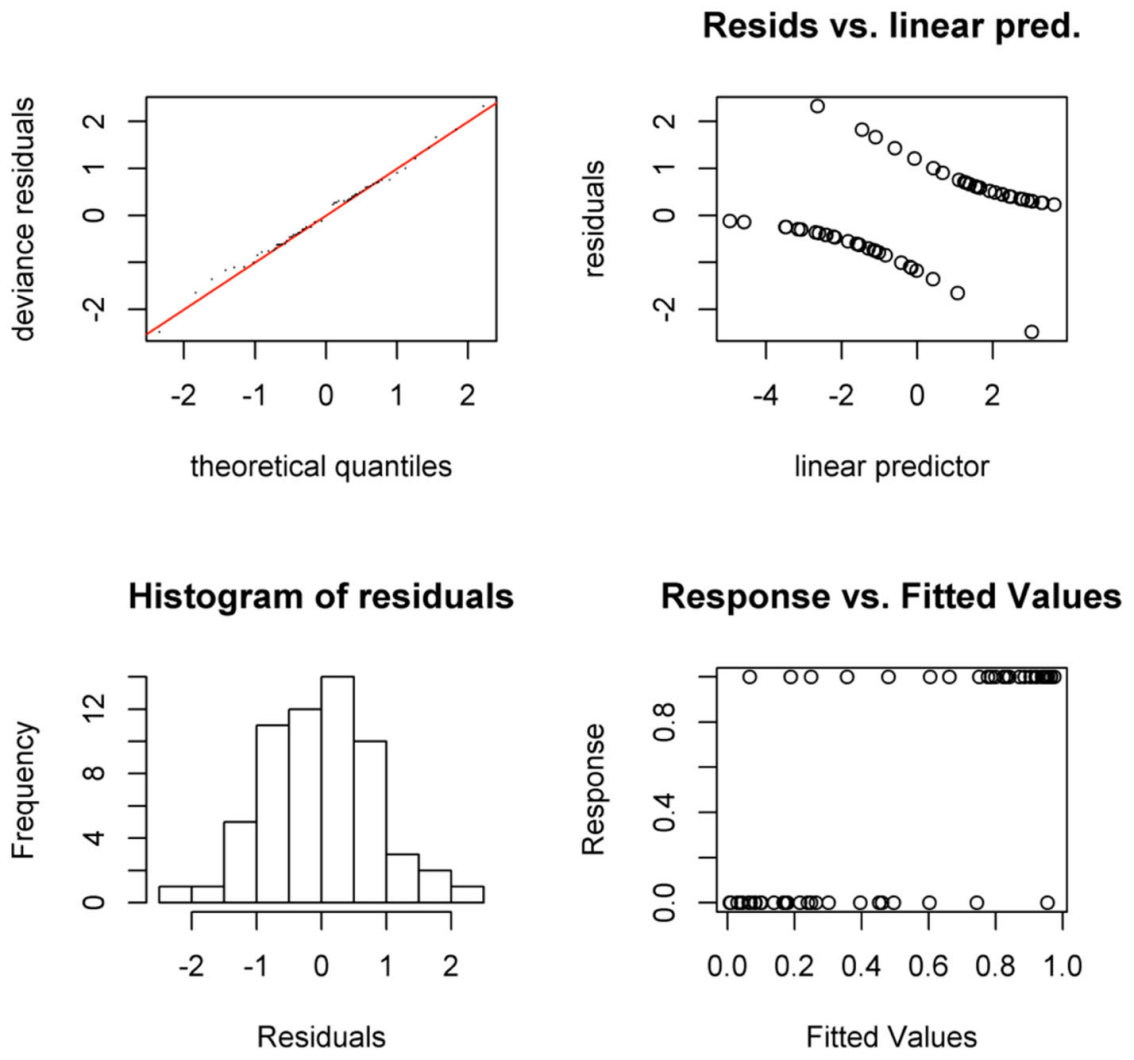
GAM Check Plots GAM plots produce deviance residuals against approximate theoretical quantilies of the deviance residual distribution. GAM: Generalized Additive Models.

These data indicate that physiological concentrations of amino acids are perturbed by *H pylori* induced PUD, and combinations of citrulline, histidine, lysine and arginine can be used to predict PUD using nonparametric modeling. The residuals for modeling GAM fitting provide information for modeling checking in the GAM check plot of Figure 5.

## Conclusion

We evaluated parametric and non-parametric modeling approaches (GAMs, Bayesian GAMs and MARS) for discovering specific physiological free amino acids as biomarkers for *H. pylori*-associated peptic ulcer disease. These studies may have potential benefits by providing non-invasive identification of individuals at risk for clinically significant ulceration and for institution of appropriate targeted therapy. This study also suggests host-interaction pathways (amino acids) related to the pathogenesis of peptic ulcer in *H. pylori* infected patients. Interestingly, in Crohn’s disease, a gastrointestinal inflammatory disease, serum citrulline was found to be inversely correlated with each other [16]. The presence of amino acids that correspond to the urea cycle could reflect the presence of a urea cycle and a highly active urease in *H. pylori*. However, differences the urea cycle amino acids in individuals who are infected but do not have PUD versus those that have PUD are not yet established, but could reflect differences in infected individuals, the infecting strains or both. We recognize that our study is limited because of the relatively small data set. Further evaluation of this modeling procedure on a large independent data set is needed.

## Figures and Tables

**Table 1:** Amino acid measurements in subjects with and without peptic ulcers.

Characteristic	Without Ulcer = 30 (50%)	With Ulcer = 30 (50%)	All subjects = 60
**Taurine**	0.15 ± 0.04	0.19 ± 0.08	0.17 ± 0.07*
**Phosphoserine**	1.3 ± 0.49	1.43 ± 0.84	1.36 ± 0.69
**Urea**	42.29 ± 11.41	54.17 ± 22.42	48.23 ± 18.63*
**Aspartic Acid**	1.39 ± 0.58	1.46 ± 0.6	1.43 ± 0.59
**Threonine**	1.85 ± 0.48	2.02 ± 0.52	1.94 ± 0.5
**Serine**	2.49 ± 0.63	2.69 ± 0.81	2.59 ± 0.73
**Glutamic Acid**	7.04 ± 1.78	7.92 ± 1.69	7.48 ± 1.78
**Glycine**	3.97 ± 0.64	4.64 ± 1.09	4.31 ± 0.95*
**Alanine**	5.74 ± 1.53	5.77 ± 2.01	5.76 ± 1.77
**Citrulline**	0.34 ± 0.1	0.54 ± 0.14	0.44 ± 0.16 §§
**Alpha Aminobutyric Acid**	0.19 ± 0.07	0.21 ± 0.08	0.2 ± 0.07
**Valine**	2.89 ± 0.69	2.94 ± 0.8	2.91 ± 0.74
**Isoleucine**	0.9 ± 0.25	1 ± 0.3	0.95 ± 0.28
**Leucine**	2.59 ± 1.08	2.69 ± 1.34	2.64 ± 1.21
**Tyrosine**	0.88 ± 0.3	0.9 ± 0.32	0.89 ± 0.31
**Phenylalanine**	1.25 ± 0.31	1.37 ± 0.39	1.31 ± 0.36
**NH3**	2.95 ± 0.59	2.95 ± 0.88	2.95 ± 0.74
**Ornithine**	1.09 ± 0.36	1.39 ± 0.74	1.24 ± 0.6
**Lysine**	2.95 ± 1	3.23 ± 1.7	3.09 ± 1.39
**Histidine**	0.97 ± 0.28	0.94 ± 0.31	0.95 ± 0.29
**Arginine**	3.21 ± 1.47	3.2 ± 1.34	3.21 ± 1.39
**Methyl Histidine1**	0.07 ± 0.06	0.1 ± 0.09	0.09 ± 0.08
**Tryptophan**	0.02 ± 0.03	0.04 ± 0.07	0.03 ± 0.05
**Carnosine**	0.09 ± 0.12	0.14 ± 0.17	0.12 ± 0.15
**Methyl Histidine3**	0.02 ± 0.02	0.03 ± 0.03	0.02 ± 0.02*
**Phosphethanolamine**	0.02 ± 0.02	0.03 ± 0.06	0.03 ± 0.05
**Beta Aminoisobutyric Acid**	0.05 ± 0.08	0.03 ± 0.05	0.04 ± 0.07
**Sarcosine**	0.07 ± 0.09	0.11 ± 0.18	0.09 ± 0.14

* P<0.05

§§ P<0.001

**Table 2:** Sparse penalized logistic regression coefficients.

Characteristic	LASSO	EN	SCAD
**Taurine**	0	1.204	0
**Phosphoserine**	0	0	0
**Urea**	0	0	0
**Aspartic Acid**	0	0	0
**Threonine**	0	0	0
**Serine**	0	0	0
**Glutamic Acid**	0	0	0
**Glycine**	0	0.137	0
**Alanine**	0	0	0
**Citrulline**	7.38	6.649	9.092
**Alpha Aminobutyric Acid**	0	0	0
**Valine**	0	0	0
**Isoleucine**	0	0	0
**Leucine**	0	0	0
**Tyrosine**	0	0	0
**Phenylalanine**	0	0	0
**NH3**	0	−0.019	0
**Ornithine**	0	0	0
**Lysine**	0	0	0
**Histidine**	0	−0.545	0
**Arginine**	0	0	0
**Methyl Histidine1**	0	0	0
**Tryptophan**	0	0	0
**Carnosine**	0	0.518	0
**Methyl Histidine3**	0	4.491	0
**Phosphethanolamine**	0	0	0
**Beta Aminoisobutyric Acid**	0	0	0
**Sarcosine**	0	0	0

**Table 3:** Model analysis of deviance tests. Two GAM analyses are shown.

GAM analysis incorporating both linear and smoothing components.			
Parameters	df	Chi-square	Pr> chisq
**Linear(Citrulline)**	1	14.4400	0.0004**
**Linear(Lysine)**	1	1.040	0.315
**Linear(Histidine)**	1	2.280	0.139
**Linear(Arginine)**	1	0.078	0.784
**Spline(Citrulline)**	2	8.596	0.014**
**Spline(Lysine)**	2	0.145	0.930
**Spline(Histidine)**	2	6.284	0.043**
**Spline(Arginine)**	2	2.141	0.343
**GAM analysis incorporating only linear components.**			
**Linear(Citrulline)**	1	13.0320	0.0007**
**Linear(Lysine)**	1	0.476	0.494
**Linear(Histidine)**	1	1.638	0.205
**Linear(Arginine)**	1	0.0001	0.989

For each, the DF, degrees of freedom and dominant factors significant at the level alpha=0.1 (*) and 0.05(**) are shown for each parameter.

**Table 4:** Marginal posterior inclusion probability and term importance. Shown are the posterior model probabilities from the MCMC 8000 samples from 8 chains, each ran 5000 iterations after a burn-in of 500.

Coefficients	P(gamma=1)	Pi	Dimension
**Linear(Citrulline)**	0.499	0.657	1*
**Spline(Citrulline)**	0.750	0.336	8**
**Linear(Lysine)**	0.006	0.000	1
**Spline(Lysine)**	0.006	0.000	6
**Linear(Histidine)**	0.026	−0.003	1
**Spline(Histidine)**	0.212	0.010	7
**Linear(Arginine)**	0.016	−0.001	1
**Spline(Arginine)**	0.015	0.000	7

* :P(gamma=1)>.25;

** :P(gamma=1)>.5

**Table 5:** Confusion matrix for the MARS model.

Class	Total	Prediction	
		H.Pylori	PUD
		(n=27)	(n=33)
**H.Pylori**	30	26	4
**PUD**	30	1	29
**Total**	60	correct = 86.67%	correct = 96.67%

**Table 6:** MARS Basis Functions. Shown are the basis functions (BF) for the MARS model of PUD prediction. Bm, each individual basis function, a_m_, coefficient of the basis function.

B_m_	Definition	a_m_	Variable descriptor
**BF4**	0.7615 −Histidine	3.49646	Histidine
**BF6**	Citrulline −0.338	6.6434	Citrulline
**BF10**	Citrulline −0.49	−7.00747	Citrulline
**BF13**	Lysine −1.401	0.121036	Lysine
**BF18**	Arginine −2.719	−0.104259	Arginine

(y)_+,_ = max(0,y)
